# Comparison of a New Radiographic Technique with MRI Measurements for Tibial Tunnel Evaluation in ACL Reconstruction

**DOI:** 10.3390/diagnostics15101237

**Published:** 2025-05-14

**Authors:** Mücahid Osman Yücel, Raşit Emin Dalaslan, Sönmez Sağlam, Zekeriya Okan Karaduman, Mehmet Arıcan, Bedrettin Akar, Volkan Tural

**Affiliations:** 1 Department of Orthopaedics and Traumatology, Faculty of Medicine, Duzce University, 81620 Duzce, Türkiye; 2 Department of Orthopedics and Traumatology, Sakarya Yenikent State Hospital, 54290 Sakarya, Türkiye; 3 Department of Orthopedics and Traumatology, Usak Training and Research Hospital, 64100 Usak, Türkiye

**Keywords:** anterior cruciate ligament reconstruction, angle measurement, tibia tunnel, radiography, sports medicine

## Abstract

**Background/Objectives**: The correct angular placement of the tibial tunnel is crucial to ensure graft tension, maintain knee stability, and ensure optimal clinical outcomes after anterior cruciate ligament (ACL) reconstruction. While 3D imaging methods such as MRI and CT are the gold standard for evaluating tunnel positioning, their routine use is limited by cost, availability, and time constraints. In clinical practice, 2D radiographs are more accessible but lack established reliability in accurately estimating tunnel angles. The aim of this study was to convert 2D radiographic angular measurements used in the evaluation of patients undergoing anterior cruciate ligament reconstruction into 3D values with a simple method and to compare these measurements with three-dimensional angles calculated using conventional MRI and CT. **Methods**: This retrospective study included 38 patients who underwent anatomic anterior cruciate ligament reconstruction. Postoperative radiographs and MR images were analyzed to determine the tibial tunnel angles. The angles calculated from 2D radiographs were statistically analyzed for their correlation with the actual 3D angles measured by MRI. **Results**: The analysis showed a strong correlation between tibial tunnel angles from radiographs and MRI, with minimal, non-significant differences. This suggests that radiographs can provide a reliable estimate of tibial tunnel angles. **Conclusions**: These findings suggest that radiographs can predict tibial tunnel angles in ACL reconstruction as accurately as MRI. This method can guide the correct tunnel angle and facilitate postoperative evaluation. Further studies are needed to confirm these results across various populations and techniques.

## 1. Introduction

Anterior cruciate ligament (ACL) injuries are a leading cause of knee instability, as the ACL prevents forward tibial shift and controls rotational stresses; when it ruptures, the risk of meniscal and additional ligament damage increases [1,2]. Surgical reconstruction reliably restores knee anatomy and function, while non-surgical treatment often leaves patients with persistent instability, reduced mobility, and difficulty returning to sports or daily activities [3,4,5].

Anterior cruciate ligament reconstruction (ACLR) has become a widely accepted and frequently performed orthopedic procedure, utilizing various surgical techniques tailored to restore the ligament’s anatomical position and mechanical function [6,7]. Among these techniques, the transtibial and anteromedial portal methods have received significant attention. A substantial body of research has emphasized that the surgical outcome of ACL reconstruction is heavily influenced by the accuracy of tibial tunnel placement and the angle at which the tunnel is drilled [7,8,9,10,11,12,13,14,15,16]. Numerous studies have reported a variety of optimal tibial tunnel angle values, each with specific clinical implications. For instance, coronal plane tunnel angles ranging from 65° to 70° have been linked to reduced flexion loss and decreased anterior laxity. However, angles exceeding 75° have been associated with significantly higher incidences of postoperative complications, such as persistent laxity and restricted motion [11]. Similarly, angles between 55° and 65° are known to promote effective bone graft healing by distributing postoperative mechanical stresses more evenly and thereby minimizing the risk of tunnel widening or graft failure [12]. As such, the precise intraoperative assessment and optimization of the tunnel angle are essential to achieve the desired surgical outcomes and minimize postoperative complications.

The literature also provides extensive evidence regarding the utility of imaging modalities, particularly roentgenograms and magnetic resonance imaging (MRI), in evaluating tibial tunnel positioning [17,18]. Although MRI is widely used in the diagnosis of ACL injuries, its diagnostic accuracy and overall role in treatment planning remain subjects of ongoing debate. Recent studies have emphasized that clinical examination may, in some cases, be more sensitive than MRI in detecting ACL ruptures and should continue to play a central role in initial assessment and treatment decision-making. As such, the preference for clinical evaluation combined with simpler, low-cost imaging modalities is increasingly supported in the literature [19,20,21,22].

While most existing studies rely on two-dimensional measurements derived from roentgenograms, it is important to note that these values only reflect the projected appearance of the three-dimensional tunnel orientation and do not provide a true spatial representation of the tunnel’s angulation [6,8,11,23,24]. Accurately capturing the true three-dimensional geometry of the tunnel generally necessitates advanced imaging techniques such as MR or computed tomography (CT) scans [9,10,13,14,25]. Although MRI is considered the gold standard, its high cost, lengthy scanning time, limited accessibility in many centers, and the requirement for advanced software and experienced personnel pose challenges for routine clinical use. In contrast, conventional radiography is a low-cost, fast, and widely accessible method. When used intraoperatively with appropriate infrastructure and fluoroscopic guidance, it can also assist in evaluating the three-dimensional placement of the tunnel. Furthermore, postoperative radiographs, which are routinely obtained in many clinics, may offer a practical advantage by enabling the more comprehensive analysis of the tunnel angle and position through this approach.

Interestingly, previous studies have demonstrated that 3D evaluations of knee kinematics can be conducted using bidirectional knee X-rays acquired through fluoroscopic imaging, suggesting a promising alternative for assessing the tunnel orientation with less complexity and cost [26,27].

The primary objective of this study is to evaluate the accuracy and clinical applicability of a geometric method that estimates three-dimensional tibial tunnel angles using standard two-dimensional knee radiographs in patients who have undergone anatomical ACL reconstruction. By comparing the radiographic angle estimations with those obtained directly from MRI, this study aims to determine whether radiography can serve as a practical and reliable alternative for postoperative tunnel evaluation in ACL reconstruction. The findings may support the development of more accessible, time-efficient, and cost-effective strategies for both intraoperative guidance and postoperative assessment in orthopedic surgery.

## 2. Materials and Methods

This retrospective study was carried out at a single orthopedic surgery center and involved patients who underwent anteromedial ACLR between January 2020 and December 2023. All procedures were performed using a standardized surgical technique in which a hamstring autograft was employed. For every case, the tibial guide angle was consistently set at 55°, in accordance with the surgical protocol used at the institution. Prior to the commencement of this research, ethical approval was obtained from the institutional ethics committee, ensuring that the study met all necessary ethical standards. The approval was officially granted on 19 August 2024, under the decision number 2024/164, confirming that the study conformed to applicable ethical guidelines and regulations.

The study cohort consisted of 38 patients who had sustained isolated anterior cruciate ligament injuries limited to a single limb. All patients included in the final analysis had undergone postoperative three-dimensional MRI and had appropriate anteroposterior (AP) and lateral knee roentgenograms available for evaluation. As the design of this research was retrospective in nature, the sample size was not determined by a power analysis prior to data collection but was instead based on the number of eligible patients meeting the inclusion criteria during the specified time frame.

To be eligible for the study, patients had to meet several key inclusion criteria: they had to be between 20 and 50 years of age, have undergone ACL reconstruction with an anteromedial portal technique, and possess sufficient high-quality postoperative imaging, including both roentgenographic and MRI data. Specifically, only postoperative AP knee roentgenograms taken in full extension with tibiofibular overlap in the range of 5 to 15 mm were considered acceptable. Additionally, lateral knee roentgenograms had to be taken in 30 to 40 degrees of knee flexion and show less than 5 mm of femoral condyle overlap in order to ensure imaging consistency and measurement reliability [7].

Patients were excluded from the study if they had undergone any previous surgical procedures for tibial fractures, as such interventions could alter their bone morphology and affect imaging interpretation. Likewise, those with periarticular implants or foreign materials that could potentially distort or obscure MRI images were not included in the analysis. Moreover, patients with incomplete medical records or missing preoperative or postoperative follow-up data were excluded, as these cases could compromise the accuracy and completeness of the study’s findings.

Throughout the course of the research, all patient information, including medical records and personal identifiers, was handled with the utmost confidentiality. In accordance with ethical standards and data protection regulations, patient identities were anonymized and not disclosed at any stage of the study. Even in the event of publication, no identifiable personal data were revealed, thereby ensuring full compliance with ethical and legal requirements concerning patient privacy.

Although this study provides valuable preliminary data, its retrospective design presents inherent limitations, including potential selection bias and unmeasured confounding factors. The patients included were those who met the strict inclusion criteria within a defined time frame, and therefore, no randomization or power analysis was performed. As a result, the findings should be interpreted with caution, and further prospective studies with larger sample sizes are recommended to confirm the generalizability and reproducibility of the results.

### 2.1. Imaging and Radiographic Measurements

In the postoperative evaluation phase, both knee roentgenograms and magnetic resonance imaging (MRI) scans of patients who had undergone ACLR and met the predetermined inclusion criteria were analyzed retrospectively. These radiographic and MRI assessments were conducted using standardized imaging protocols to ensure the accuracy and comparability of measurements. The image analyses and angular measurements were independently performed by two experienced observers, both of whom were orthopedic surgeons with subspecialty training in traumatology. Their evaluations were conducted in a blinded fashion to minimize observer bias and ensure objective data collection.

**Figure 1:**
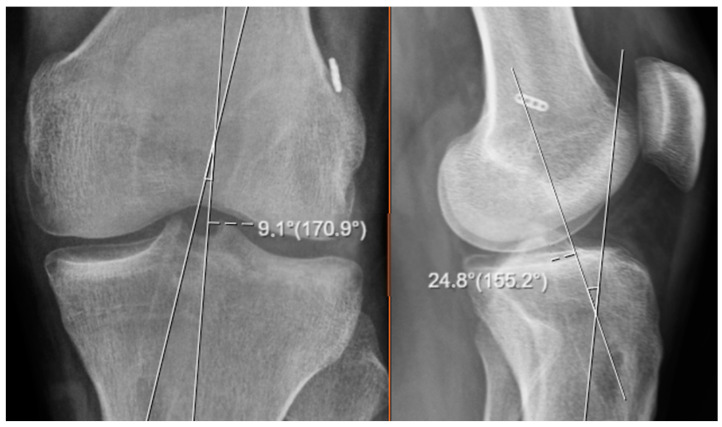
Image showing the measurement of tunnel angles on knee roentgenograms.

For each patient, angular measurements were obtained from both anteroposterior (AP) and lateral knee roentgenograms. On these radiographs, the angle formed between the anatomical longitudinal axis of the tibia and the axis of the tibial tunnel was measured separately for each imaging plane. These two-dimensional angular values were subsequently utilized in a geometric formula to calculate the three-dimensional spatial orientation of the tibial tunnel. The purpose of this process was to estimate the actual tunnel position in a three-dimensional space based solely on the information obtained from standard two-plane radiographic imaging (see Figure 1).

**Figure 2:**
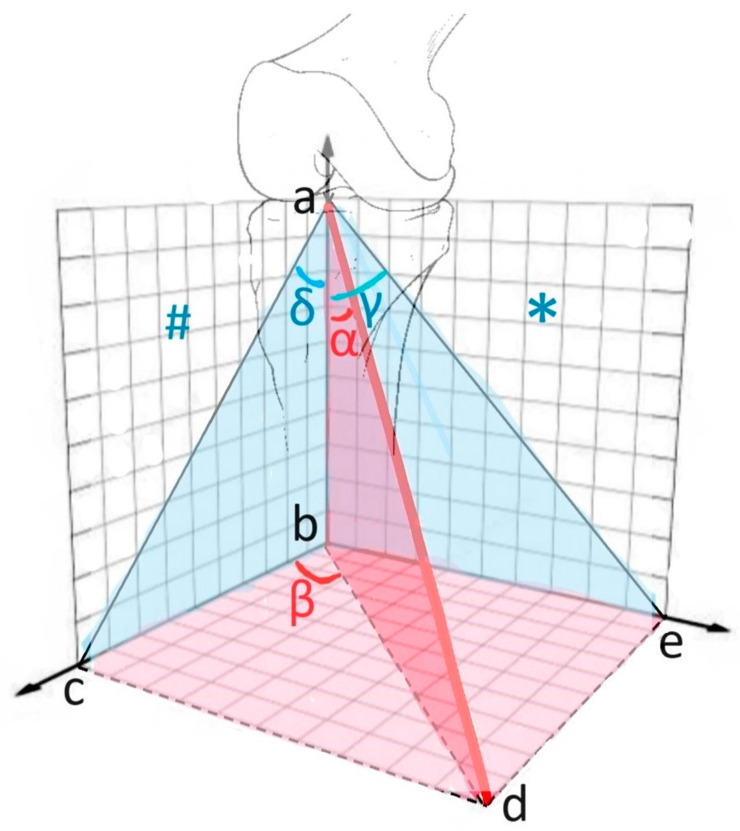
Illustration showing the planes and shapes in which the calculations were performed; |ab| = anatomical axis of the tibia = vertical axis, |ad| = axis of the tibial tunnel, ∗ = coronal plane = plane of the AP knee roentgenogram, γ angle = angle between the tibial tunnel and the anatomical axis of the tibia measured on the AP knee roentgenogram, # = sagittal plane = plane of the lateral knee roentgenogram, δ angle = the angle measured between the tibial tunnel and the anatomical axis of the tibia measured on the lateral knee roentgenogram, α angle = the angle between the tibial tunnel and the anatomical axis of the tibia, β angle = the angle between the plane of the tibial tunnel and the sagittal plane.

In terms of the anatomical orientation, the AP knee roentgenogram was defined as corresponding to the coronal plane, which provides a frontal view of the knee, while the lateral knee roentgenogram was regarded as representative of the sagittal plane, offering a side profile of the joint. All calculations for the three-dimensional angle estimation were based on these respective planes. This methodological approach allowed the study to simulate a 3D angular perspective using only conventional 2D radiographic images, providing an efficient and accessible alternative to more advanced imaging modalities (see Figure 2).

### 2.2. Geometric Calculations

The angles α, β, indicating the 3D position of the tibial tunnel, were calculated using geometric methods. For example, if we assume that |ab|, which represents the anatomical axis of the tibia, is 1 cm, we obtain the following results:

$$tan(\alpha )=\frac{\left|bd\right|}{\left|ab\right|}=|bd|$$

$$tan(\gamma )=\frac{\left|be\right|}{\left|ab\right|}=|be|$$

$$tan(\delta )=\frac{\left|bc\right|}{\left|ab\right|}=|bc|$$

If we calculate over the right triangle (bcd) with the help of these results, and if we substitute the data we obtained in the equations ${\left|bd\right|}^{2}={\left|bc\right|}^{2}+{\left|cd\right|}^{2}$ and $tan\left(\beta \right)=\frac{\left|cd\right|}{\left|bc\right|}$, we obtain the following equations:

$$tan{\left(\alpha \right)}^{2}=tan{\left(\delta \right)}^{2}+tan{\left(\gamma \right)}^{2}\to \alpha =arctan\sqrt{tan{\left(\delta \right)}^{2}+tan{\left(\gamma \right)}^{2}}$$

$$tan(\beta )=\frac{tan(\gamma )}{tan(\delta )}\to \beta =arctan\frac{tan(\gamma )}{tan(\delta )}$$

The angles γ and δ, which were initially and directly measured from standard knee roentgenograms, served as the foundational input values in the geometric equations utilized for the three-dimensional calculations. These simple two-dimensional angular measurements were integrated into a set of mathematical formulas designed to derive the more complex angles α and β, which represent the actual three-dimensional orientation of the tibial tunnel. In order to perform these calculations accurately and efficiently, the Microsoft Math Solver software (version available as of January 2025) was employed. This digital tool enabled the rapid and precise execution of the trigonometric computations required for the spatial analysis, facilitating consistent results across all cases included in the study.

**Figure 3:**
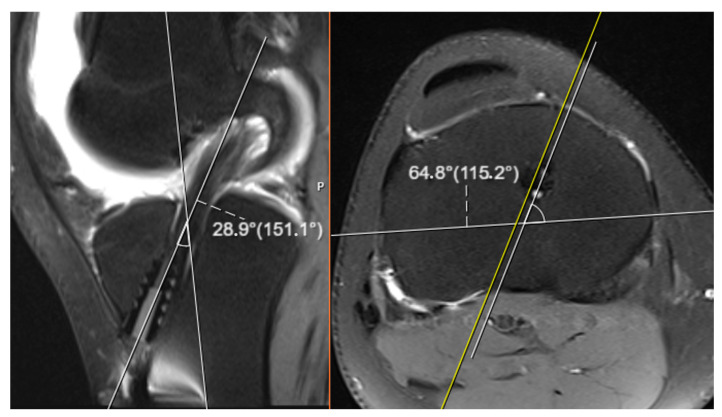
Image showing the measurement of tunnel angles on MRI.

The tibial tunnel angles derived through these three-dimensional calculations obtained solely from conventional roentgenographic images were subsequently compared with the actual tunnel angles measured directly on magnetic resonance imaging (MRI) scans. This comparison was performed to evaluate the degree of correlation, the extent of deviation, and the overall accuracy between the two distinct measurement approaches (see Figure 3). In the context of this study, the tunnel angles computed from roentgenograms were denoted by the symbols α and β, whereas the corresponding angles measured on MRI images were labeled as α′ (alpha prime) and β′ (beta prime), respectively. This symbolic differentiation was used throughout the analysis to clearly distinguish between values obtained by calculation and those determined via imaging.

For the MRI-based evaluations, oblique slices oriented parallel to the axis of the tibial tunnel were utilized in order to achieve accurate three-dimensional representations. These specific oblique images were carefully selected to align with the true orientation of the tunnel, allowing for more reliable angle measurement. In each case, two principal angular relationships were assessed: the angle formed between the tibial tunnel and the sagittal axis of the knee joint, and the angle formed between the tunnel and the anatomical longitudinal axis of the tibia. These MRI-based angle measurements served as the reference standard for assessing the validity and precision of the radiograph-based three-dimensional calculations.

### 2.3. Statistical Data Analysis

The data obtained as a result of the research were transferred to the computer environment and edited with the Microsoft Excel package program and then analyzed with the SPSS (Statistical Package for Social Sciences) 29.0 package program. Before starting the analysis, the conformity of the numerical data to normal distribution was examined by Kolmogorov–Smirnov, Shapiro–Wilk, skewness and kurtosis tests, and Histogram and Q-Q Plot graphs. The normality results for the data are given in Table 1. The measurements of 38 participants from two different devices were analyzed with an Independent Sample *t* Test and the significance value was accepted as *p* < 0.05. Interobserver agreement between the two orthopedic surgeons was assessed using the intraclass correlation coefficient (ICC). A two-way random effects model with absolute agreement was applied. ICC values greater than 0.75 were considered to indicate good agreement.

## 3. Results

The analysis reveals a strong agreement between the angles measured by radiographs and 3D MRI. The mean differences between the angles measured by the two modalities were minimal and not statistically significant. This suggests that simple knee radiographs can be used as a reliable alternative to MRI measurements to calculate angles.

According to the Kolmogorov–Smirnov and Shapiro–Wilk tests, a *p*-value above 0.05 indicates that the data are normally distributed. Additionally, based on the criterion defined by Tabachnick and Fidell (2013) [28], skewness and kurtosis values between −1.5 and +1.5 further confirm normality. As shown in Table 1, all α and β angles measured by both MRI and radiograph meet these conditions, indicating that the data are suitable for parametric testing.

The results of the independent samples *t*-test comparing MRI and radiographic measurements are presented in Table 2. The mean differences between the two modalities were minimal for both α and β angles, and the *p*-values (0.842 and 0.894, respectively) were far from statistical significance. These findings demonstrate a strong agreement between the two measurement techniques.

**Figure 4:**
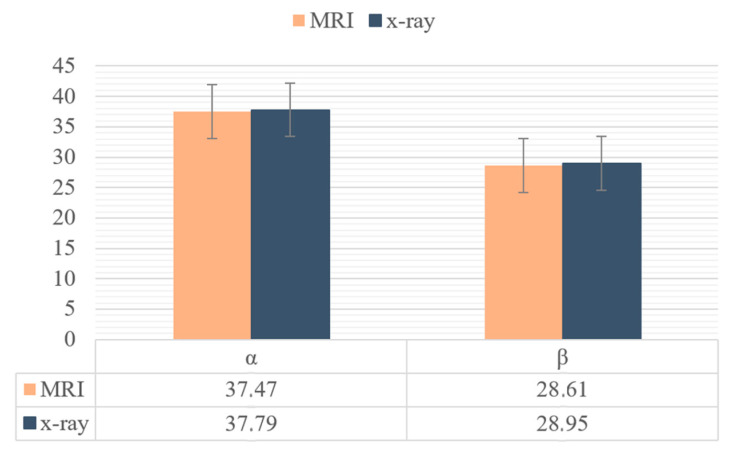
Graphical representation of mean angle values.

Therefore, the study suggests that radiographic measurements, when calculated appropriately, can provide reliable estimates of tibial tunnel angles comparable to those obtained with MRI. Figure 4 illustrates the mean values of each angle, visually reinforcing the close alignment between the methods.

**Figure 5:**
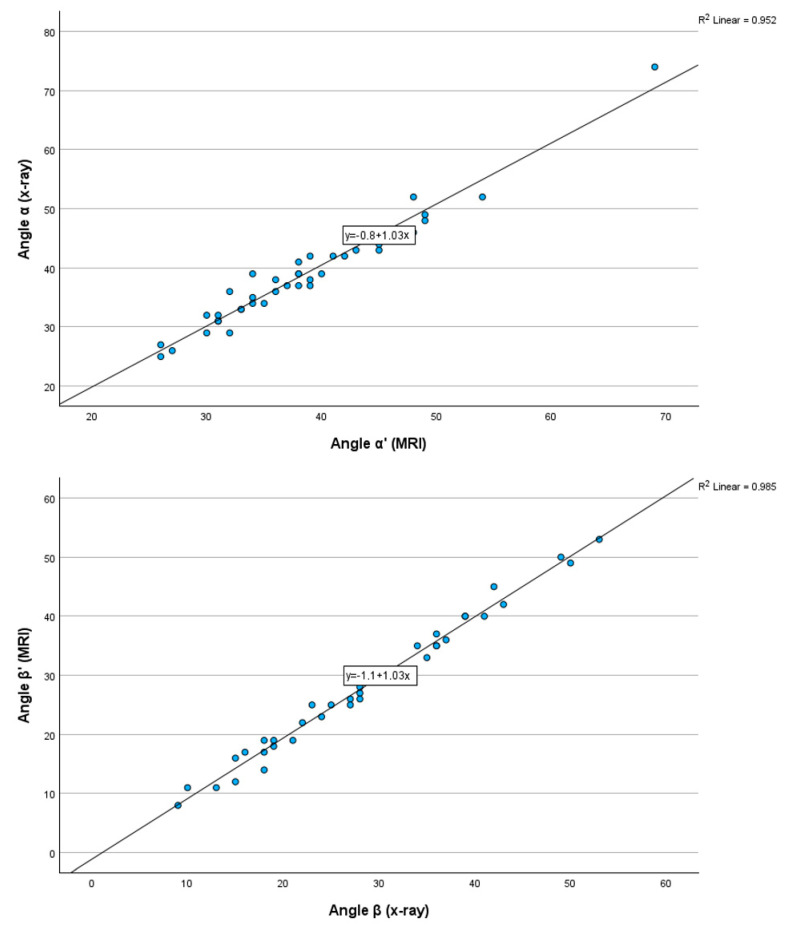
Graphs showing angle values on a scatter plot in MRI and X-ray.

These results show that the tibial tunnel angles measured by MR are highly correlated with those measured on roentgenograms and that MR images are a reliable alternative for assessing tibial tunnel angles. Figure 5 shows a graphical representation of the angle values on a scatter plot.

The statistical findings of this study demonstrate that radiographic measurements show a high degree of consistency with MRI-based assessments of tibial tunnel angles. This result supports the notion that radiographs can serve as a reliable alternative for evaluating tunnel orientation. Moreover, the geometric method applied to conventional radiographs appears to have sufficient precision to allow the accurate intraoperative estimation of tunnel position when used with fluoroscopy, especially in settings where advanced imaging is not readily available. In the postoperative period, this approach may also enable a more detailed radiographic analysis of tunnel alignment, providing surgeons with a practical, low-cost tool that can contribute to improved surgical planning, follow-up, and clinical decision-making.

## 4. Discussion

In this study, we calculated the tibial tunnel angles of patients with anatomic ACLR using three-dimensional methods on roentgenograms and compared these angles with the actual angles measured by MRI. The results of our study show a strong agreement between the radiographically calculated tibial tunnel angles and the actual angles measured by MR imaging. This finding suggests that simple roentgenograms may be a useful alternative for assessing tibial tunnel angles.

The measurement of tibial tunnel angulation has been evaluated in many studies. For example, in a study by Zhang et al., the mean beta angle was 42.8° ± 3.4° and the mean alpha angle was 45.3° ± 5.1° in patients undergoing ACLR [10]. In our study, we found an alpha angle of 37.47° ± 6.86° and a beta angle of 28.61° ± 11.34° on MRI, while the alpha angle was 37.79° ± 6.89° and the beta angle was 28.95° ± 11.02° on radiogram. In our study, we set the tibial guide at 55°, which indicates that the alpha angle should be 35°; we obtained a result close to this value, with 37°. Notable discrepancies exist between the angular values reported in previous studies [10,11,12,13,14,15,16], which we believe are primarily due to differences in surgical techniques, the intraoperative tibial guide settings, and tunnel orientation preferences. Given the biomechanical and clinical consequences of these variations, this highlights the importance of the intraoperative standardization of the tibial tunnel angle to improve consistency and surgical outcomes. Our study contributes to the literature by proposing a simplified, reproducible method that can assist in achieving this goal using routine radiographic assessment.

Different studies have made various recommendations for tibial tunnel angles. Youm et al. suggested an alpha angle of 45°, while Chang et al. suggested a tibial guide of 50°, i.e., an alpha angle of 40° [14,15]. Zhao et al. reported that the alpha angle should be 50° and the beta angle 40° [13]. A biomechanical study by Pena et al. using computer simulations concluded that an alpha angle of 40° reduces the stress load on the menisci and that a decrease in this angle increases the load on the meniscus [16]. Howell et al. recommended avoiding angles of 75° or greater in the coronal plane (γ angle less than 15°) because these angles both increase the range of motion lost and lead to laxity [11]. Furthermore, according to Yao et al., tibial guide angles in the range of 55–65°, i.e., an alpha angle of 25–35°, promote bone graft compatibility by balancing stress distribution postoperatively and reduce the risk of tunnel enlargement [12]. Therefore, careful planning of the tibial tunnel angle is critical to reduce the risks of postoperative complications and ensure the optimal biomechanical fit of the graft. These findings collectively emphasize that deviations in the tunnel angle can significantly influence graft biomechanics, joint stability, and postoperative outcomes. Therefore, the accurate and reproducible intraoperative assessment of tibial tunnel angle is essential. In this context, our study offers a clinically applicable method to support precise tunnel orientation using accessible imaging tools.

Although 3D CT and 3D MRI are frequently used in the literature to evaluate the 3D position of the tibial tunnel, the more commonly preferred roentgenograms are 2D and therefore fail to accurately reflect this position [29]. Behrend et al. measured the 2D γ angle, which was also used in our study, and found an average of 24° [24]. Similarly, researchers such as Júnior et al., Simmons et al., and Sharma et al. used the γ angle obtained from AP knee roentgenograms [7,8,23]. Howell et al. (2001) recommended that the γ angle should be greater than 15° [11]. However, since these measurements are made in a two-dimensional plane, they may not accurately reflect the tibial tunnel position and these measurements using only AP knee roentgenograms may be insufficient. The method used in our study offers a key advantage in this regard, as it allows for the estimation of the tunnel’s three-dimensional orientation by combining measurements from both AP and lateral radiographs using a geometric approach.

In the existing literature, most studies have relied on 2D radiographs to evaluate tibial tunnel angles. However, these methods are inherently limited in their ability to describe the true 3D spatial orientation of the tunnel. Although some researchers have used advanced imaging techniques such as CT and MRI to obtain more accurate assessments, these modalities are often constrained by high costs, limited accessibility, and longer processing times. In contrast, the method proposed in our study enables the 3D evaluation of tunnel positioning using standard radiographs. This simplified yet effective approach has the potential to support surgeons both intraoperatively and postoperatively by offering a practical and accessible tool for tunnel angle assessment.

This study has several limitations. First, its retrospective design may introduce biases related to data collection and missing information. The relatively small sample size (*n* = 38) and lack of a power analysis limit the statistical strength and generalizability of the findings. Additionally, the single-center design may reflect a narrow range of surgical techniques and patient characteristics, reducing external validity. To address these limitations, future prospective and multicenter studies with larger and more diverse patient populations are needed to confirm our results and evaluate their impact on long-term clinical outcomes.

## 5. Conclusions

This study demonstrates that standard knee radiographs can be used as a practical and reliable tool for evaluating tibial tunnel angles, offering a feasible alternative to MRI and CT, which are often limited by cost, accessibility, radiation exposure, and longer processing times. The geometric method presented here, based on routinely obtained anteroposterior and lateral radiographs, may assist surgeons in both intraoperative tunnel orientation and postoperative evaluation.

Furthermore, this study demonstrates that it is possible to extract three-dimensional angular information from standard two-dimensional radiographs. This approach can also be integrated with intraoperative fluoroscopy, potentially simplifying other future surgical procedures.

However, the results of this study should be validated in larger, prospective, multicenter trials. Future research should focus on evaluating the applicability of this method across different surgical techniques, its reproducibility in various patient populations, and its correlation with long-term clinical outcomes. Moreover, the integration of this technique into digital imaging systems or semi-automated platforms may improve both its accuracy and ease of use in routine orthopedic workflows.

## Figures and Tables

**Table 1:** Results of the normality analysis.

		Kolmogorov-Smirnov			Shapiro-Wilk			Skewness	Kurtosis
		Statistic	df	Sig.	Statistic	df	Sig.		
Angle	α′ (MRI)	0.096	38	0.200	0.971	38	0.419	0.424	−0.316
	α (radiogram)	0.088	38	0.200	0.982	38	0.795	0.208	−0.418
Angle	β′ (MRI)	0.091	38	0.200	0.979	38	0.693	0.282	−0.555
	β (radiogram)	0.080	38	0.200	0.979	38	0.670	0.268	−0.505

**Table 2:** Analysis of the difference between MRI and radiogram.

	Grup	*N*	Mean	Std. Deviation	t	*p*-Value
Angle	α′ (MRI)	38	37.47	6.86	−0.200	0.842
	α (radiogram)	38	37.79	6.89		
Angle	β′ (MRI)	38	28.61	11.34	−0.133	0.894
	β (radiogram)	38	28.95	11.02		

## Data Availability

The data presented in this study are available upon request from the corresponding author. The data are not publicly available due to ethical restrictions.

## Abbreviations

The following abbreviations are used in this manuscript:

ACL	Anterior Cruciate Ligament
ACLR	Anterior Cruciate Ligament Reconstruction
AP	Anteroposterior
CT	Computed Tomography
MRI	Magnetic Resonance Imaging
3D	Three-Dimensional
2D	Two-Dimensional
PACS	Picture Archiving and Communication System
SPSS	Statistical Package for the Social Sciences
Q-Q Plot	Quantile–Quantile Plot
